# Congenic rats with higher arylamine N-acetyltransferase 2 activity exhibit greater carcinogen-induced mammary tumor susceptibility independent of carcinogen metabolism

**DOI:** 10.1186/s12885-017-3221-9

**Published:** 2017-03-31

**Authors:** Marcus W. Stepp, Mark A. Doll, David J. Samuelson, Mary Ann G. Sanders, J. Christopher States, David W. Hein

**Affiliations:** 1 Department of Pharmacology and Toxicology, School of Medicine, University of Louisville, Louisville, KY 40202 USA; 2 James Graham Brown Cancer Center, University of Louisville, Louisville, KY 40202 USA; 3 Department of Biochemistry & Molecular Genetics, School of Medicine, University of Louisville, Louisville, KY 40202 USA; 4 Department of Pathology, University of Louisville Hospital, Louisville, KY 40202 USA

**Keywords:** Human arylamine N-acetyltransferase 1 (NAT1), Rat arylamine N-acetyltransferase 2 (NAT2), Acetyl-coenzyme A (AcCoA), Chemically-induced tumorigenesis, Methylnitrosourea (MNU), 7,12-dimethylbenzanthracene (DMBA)

## Abstract

**Background:**

Recent investigations suggest role(s) of human arylamine *N*-acetyltransferase 1 (NAT1) in breast cancer. Rat NAT2 is orthologous to human NAT1 and the gene products are functional homologs. We conducted in vivo studies using F344.WKY-*Nat2* ^*rapid/slow*^ rats, congenic at rat *Nat2* for high (rapid) and low (slow) arylamine N-acetyltransferase activity, to assess a possible role for rat NAT2 in mammary tumor susceptibility.

**Methods:**

Mammary carcinogens, methylnitrosourea (MNU) and 7,12-dimethylbenzanthracene (DMBA) neither of which is metabolized by N-acetyltransferase, were administered to assess mammary tumors. MNU was administered at 3 or 8 weeks of age. DMBA was administered at 8 weeks of age. NAT2 enzymatic activity and endogenous acetyl-coenzyme A (AcCoA) levels were measured in tissue samples and embryonic fibroblasts isolated from the congenic rats.

**Results:**

Tumor latency was shorter in rapid NAT2 rats compared to slow NAT2 rats, with statistical significance for MNU administered at 3 and 8 weeks of age (*p* = 0.009 and 0.050, respectively). Tumor multiplicity and incidence were higher in rapid NAT2 rats compared to slow NAT2 rats administered MNU or DMBA at 8 weeks of age (MNU, *p* = 0.050 and 0.035; DMBA, *p* = 0.004 and 0.027, respectively). Recombinant rat rapid-NAT2, as well as tissue samples and embryonic fibroblasts derived from rapid NAT2 rats, catalyzed *p*-aminobenzoic acid *N*-acetyl transfer and folate-dependent acetyl-coenzyme A (AcCoA) hydrolysis at higher rates than those derived from rat slow-NAT2. Embryonic fibroblasts isolated from rapid NAT2 rats displayed lower levels of cellular AcCoA than slow NAT2 rats (*p* < 0.01).

**Conclusions:**

A novel role for rat NAT2 in mammary cancer was discovered unrelated to carcinogen metabolism, suggesting a role for human NAT1 in breast cancer.

## Background

Human arylamine N-acetyltransferase 1 (NAT1) is a cytosolic isoenzyme responsible for the N-acetylation of arylamine xenobiotics, including environmental and occupational carcinogens such as 4-aminobiphenyl (ABP) [1]. *NAT1* is located on the short arm of chromosome *8* (*8p22*) and is expressed in nearly all human tissues [2, 3]. NAT1 catalyzes both *O*-acetylation and *N*-acetylation. One role for NAT1 in carcinogenesis is its ability to biotransform arylamine procarcinogens to active carcinogens [4]. Recent findings in vitro suggest that NAT1 activity level also may influence cancer cell proliferation and survival [5–8].

NAT1 activity is modified by genetic polymorphism, but also can be regulated by microRNA, epigenetic, and/or translational and post-translational control features [9–12]. *NAT1* transcript levels are regulated by extracellular stimuli acting on glucocorticoid receptor or androgen receptor [13, 14]. Exposure to NAT1 substrates can increase NAT1 degradation [11, 12]. Some polymorphisms in humans destabilize the enzyme, leading to greater polyubiquitin-dependent degradation [11]. NAT1*10, the most common haplotype, is associated with increased NAT1 activity in human bladder [15], colon [16], and liver [17]. NAT1*10 has been associated with higher risk of developing cancers of the breast [18], colon/rectum [19, 20], lung [21], pancreas [22], and urinary bladder [23]. However, other studies have reported no association between NAT1*10 and cancer risk [24, 25].

Rat arylamine N-acetyltransferases are similar in sequence and function to human N-acetyltransferases [26–28]. Rat NAT2 and human NAT1 active sites both contain Phe125, Arg127, and Tyr129, consistent with their similar arylamine substrate selectivity [28]. The C-terminal undecapeptide, which is involved in controlling acetyl-coenzyme A (AcCoA) hydrolysis [29], is 100% identical when comparing rat NAT2 and human NAT1.

Fischer 344 (F344) and Wistar Kyoto (WKY) inbred rat strains have been characterized as rapid and slow NAT2 acetylators, respectively [30, 31]. Slow acetylator WKY inbred rats are homozygous for a *Nat2* allele that contains four single nucleotide polymorphisms (SNPs): G^361^A (Val^121^ → Ile), G^399^A (synonymous), G^522^A (synonymous), and G^796^A (Val^266^ → Ile), compared to the F344 strain rapid-acetylator *Nat2* allele [32]. Our laboratory constructed and characterized congenic F344.WKY rats with high (rapid) and low (slow) NAT2 activities [33].

In the current study, we utilized rapid (F344.WKY-*Nat2* ^*rapid*^) and slow (F344.WKY-*Nat2* ^*slow*^) acetylator rat strains to investigate mammary cancer risk following the administration of methylnitrosourea (MNU) or 7,12-dimethylbenzanthracene (DMBA), neither of which is biotransformed by rat NAT2 [34–37]. The results of this study provide new evidence and insight into a role of rat NAT2 and, by analogy, human NAT1 in breast cancer susceptibility.

## Methods

### Rapid and slow acetylator rats congenic at the Nat2 locus

Rapid and slow acetylator *Nat2* congenic Fischer 344 (F344.WKY) rats were housed in the University of Louisville animal facility and the experiments were reviewed and approved by the University’s Institutional Animal Care and Use Committee. The construction of rapid and slow acetylator *Nat2* congenic rats was previously reported [33]. Briefly, F344 (homozygous *Nat2* rapid) males were mated to WKY (homozygous *Nat2* slow) females to produce the obligate heterozygous F_1_ generation. F_1_ females were then backcrossed with F344 males. Heterozygous acetylator female progeny from each successive backcross were identified by rat *Nat2* genotype and were mated with F344 rapid acetylator males. After ten generations of backcrossing, heterozygous acetylator brother/sister progeny were mated to produce the homozygous rapid and slow acetylator congenic rat *Nat2* lines. The congenic F344.WKY rats have been confirmed for rapid and slow phenotypic differences in activity across multiple tissues and with different substrates [33].

### Methylnitrosourea administration

**Fig. 1:**
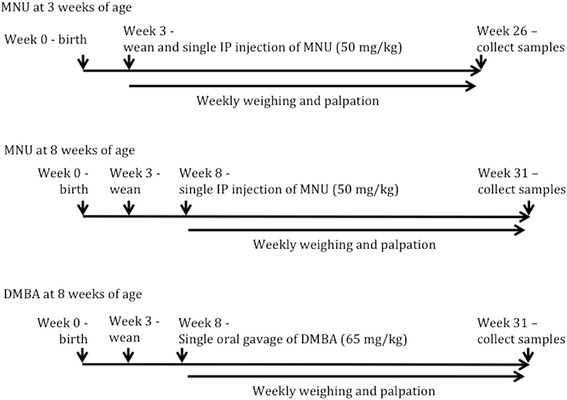
Experimental design for chemical-induced tumor experiments. Top shows the dosing of MNU at 3 weeks of age. The middle displays the dosing of MNU at 8 weeks of age. The bottom depicts the dosing of DMBA at 8 weeks of age

Forty-two female rapid acetylator congenic rats and thirty-four female slow acetylator congenic rats, at 3 weeks of age, were administered methylnitrosourea (MNU; CAS#: 684-93-5)(Ash-Stevens, Detroit, MI) by a single intraperitoneal (IP) injection of 50 mg/kg (10 mg/ml) solution dissolved in saline pH 5.0, acidified with glacial acetic acid (Fig. 1) [38–40]. Six rapid and six slow acetylator control female rats were injected with vehicle. During the twenty-three week experiment, one rapid (17 weeks) and two slow acetylator rats (18 and 18 weeks) were euthanized due to tumor burden and/or size. These data were incorporated into the statistical analysis.

Twenty-four rapid acetylator female congenic rats and thirty-three slow acetylator female congenic rats, at 8 weeks of age, were administered MNU by a single IP injection of 50 mg/kg (10 mg/ml) as above (Fig. 1). Three rapid and four slow acetylator control female rats were injected with vehicle. During the study one rapid female acetylator rat was euthanized (16 weeks) because of tumor burden and/or size. This sample was included in the statistical analysis.

### 7,12-dimethylbenzanthracene administration

Twenty-eight rapid acetylator female congenic rats and thirty-three slow acetylator female congenic rats were given a single dose of 7,12-dimethylbenzanthracene dissolved in sesame seed oil (DMBA; CAS#: 57-97-6)(Acros Organics, New Jersey, USA) (65 mg/kg) by oral gavage at 8 weeks of age (Fig. 1) [41, 42]. Three rapid and three slow acetylator control female rats were injected with vehicle. During the study four rapid female acetylator rats were euthanized (14, 17, 20, and 21 weeks) because of weight loss or tumor burden issues. Additionally six slow acetylator females were euthanized (9, 17, 14, 20, 21, and 21 weeks) for weight loss or tumor burden issues. These data were still utilized for statistical analysis.

### Weekly monitoring of animal weight and palpable mammary tumors

Rats were weighed weekly following carcinogen administration. Rats that displayed bradykinesia/fatigue, a tumor size =10% of body weight, or tumors that were ulcerated (any break in skin observed) were euthanized. Rats were palpated weekly. The number of weeks post-carcinogen exposure of first palpable mammary tumor was recorded.

### Mammary tissue and tumor collection

Twenty-three weeks after administration of a carcinogen, rats were euthanized by CO_2_ asphyxiation followed by cervical dislocation. The rats were immediately dissected to count mammary tumors that were ≥3 mm in any direction, collect samples, and record any abnormalities. Mammary tumor and adjacent mammary-tissues were removed and fixed in 10% neutral buffered formalin.

### Pathology

Tissue samples described above were processed and stained by the Special Procedure Laboratory at the University of Louisville. In brief, tissue was paraffin embedded and sections cut at 5 μm. Sections were stained using hematoxylin and eosin (H&E). H&E stained slides were evaluated and categorized, based on histomorphology, into the following groups: Normal, Benign, Low Grade *Carcinoma* In Situ *(CIS)*, Intermediate Grade *CIS*, High Grade *CIS* and Invasive Carcinoma. If a mammary tumor demonstrated multiple features of more than one category, the most malignant category was used for classification. Rats with multiple tumors were categorized by the most severe classification. These evaluations and classifications were blind to rat treatment and phenotype.

### Rapid and slow acetylator rat NAT2 recombinant expression and enzymatic activity

Recombinant rapid and slow acetylator rat NAT2 proteins were expressed in JM105 *E. coli* as described [32, 43]. Total bacterial lysate protein concentration was determined for each expression using the Bio-Rad protein assay kit (Bio-Rad, Hercules, CA). Bacterial lysates were assayed for *N*-acetylation of *para*-aminobenzoic acid (PABA) and folate-dependent AcCoA hydrolysis.

For determination of PABA *N*-acetylation activity, lysates (in triplicate) were incubated with 1 mM AcCoA and 300 μM PABA for 10 min at 37 °C, and the reaction was stopped by addition of 1/10 volume of 1 M acetic acid. The reaction tubes were centrifuged to remove precipitated protein, and supernatant was injected onto a LiChrospher 100 RP-18 (125 mm X 4 mm; 5 μm) reverse-phase column. Reactants and products were separated by high-performance liquid chromatography (HPLC)(Beckman Coulter, Fullerton, CA). *N*-Acetyl-PABA product was quantitated by its absorbance at 280 nm as described [43].

Bacterial lysates were assayed for folate-dependent AcCoA hydrolytic activity as reported [43]. In brief, lysates (in triplicate) were incubated with 500 μM AcCoA in the presence or absence of 300 μM folate for 10 min at 37 °C, and the reaction was quenched by adding 1/10 volume of perchloric acid (15% *w*/*v*). The precipitated protein was removed by centrifugation and the supernatant was injected onto a C18 reverse-phase HPLC column (250 mm X 4 mm; 5 μm). Reactants and products were separated and quantitated by HPLC. Separation of CoA, acetyl CoA, and folate was achieved using a linear gradient of 100% 55 mM sodium phosphate (NaH_2_PO4) pH 4.0: 0% methanol to 0% 55 mM sodium phosphate pH 4.0: 100% methanol over 20 mins and was quantitated by absorbance at 260 nm. The amounts of CoA produced in the minus folate reactions were subtracted from reactions containing folate to determine folate-dependent hydrolysis. Control reactions without the addition of protein lysate also were included. The limit of detection for CoA was 0.05 nmoles/min/mg protein.

### Rapid and slow congenic rat tissue lysate preparation and enzymatic activity

Liver, lung, colon, and mammary tissues were collected from 8 week old female rapid and slow NAT2 congenic rats. Tissues were homogenized in 20 mM sodium phosphate (pH 7.4), 1 mM EDTA, 0.2% triton X-100, 1 mM dithiothreitol, 100 μM phenylmethanesulfonyl fluoride, 10 μg/ml aprotinin, and 1 μM pepstatin. Homogenates were centrifuged at 15,000 x *g* for 10 min, and aliquots of supernatants were stored at −80 °C until used. Protein concentrations were determined using the Bio-Rad protein assay kit.

To measure PABA *N*-acetylation activity, reactions containing tissue lysate (<2 mg of protein/ml), 300 μM PABA and 1 mM AcCoA were incubated at 37 °C for 10 min. Reactions were terminated by the addition of 1/10 volume of 1 M acetic acid. Activity was quantified by HPLC as described above.

To measure folate-dependent AcCoA hydrolytic activity, reactions containing tissue lysate (<2 mg of protein/ml), 300 μM folate and 500 μM AcCoA were incubated at 37 °C for 10 min. Reactions were terminated by the addition of 1/10 volume of perchloric acid (15% *w*/*v*). Activity was quantified by HPLC as described above.

### Rat embryonic fibroblasts isolation

Rat embryonic fibroblasts (REFs) were isolated from congenic rats by adapting procedures described previously for mouse embryonic fibroblasts [44]. Dams were euthanized by CO_2_ asphyxiation and cervical dislocation on E13.5 (13.5 days post copulation) and embryos removed by Caesarean section. Embryonic brain tissue and organs were removed. The resultant tissue was dissociated by gentle enzymatic digestion [0.05% trypsin (*w*/*v*) for 15 mins] and cells were plated into 10 cm dishes (one embryo per dish) in DMEM (Dulbecco’s modified Eagle’s medium; high glucose, Lonza, Walkersville, MD, USA) supplemented with 10% FBS (fetal bovine serum), 2 mM L-glutamine, 100 units/ml penicillin, 100 mg/ml streptomycin and 0.25 mg/ml amphotericin. Passaging was by cell dissociation from plates [0.05% trypsin (*w*/*v*) for 15 mins] and re-plating 5 × 10^5^ cells on fresh 10 cm dishes. Resulting REF cell lines at passage 4 were characterized for their PABA N-acetylation activities as described above.

### REFs endogenous cellular AcCoA concentration measurements

Endogenous acetyl-CoA levels within REFs were determined by HPLC. On passage 4, the REFs were plated at 5 × 10^5^ per plate and allowed to grow for 96 h. After 96 h, media were aspirated from each plate, and washed with 5 ml phosphate-buffered saline (PBS). Then cells were gently dissociated from the plate with 0.5 ml trypsin [0.05% (*w*/*v*) for 15 mins]. Cells were suspended in 4 ml of diluted complete media (20% medium and 80% PBS). Aliquots of 0.5 ml were used to count the number of cells per ml. In the subsequent steps all cells and lysates were kept on ice or in 4 °C conditions. Collected cells were washed with ice cold PBS, and transferred to a 1.5 ml microcentrifuge tubes. The suspended cells were collected by centrifugation and the supernatants discarded. Having removed any residual PBS, the cells were suspended in 100 μl of ice-cold 5% 5-sulfosalicylic acid with a 1 ml BD Insulin Syringe with permanently attached 28 gauge needle (BD Franklin Lakes, NJ, USA). The cellular lysate was then centrifuged at 13,000 x g for 10 min. Supernatant was filtered through a syringe filter (13 mm, 0.20 μm pore size). Filtrate was collected and separated on a C18 reverse-phase HPLC column (250 mm × 4 mm; 5 μm pore size). HPLC separation and quantitation of AcCoA was achieved using the same method described above for folate-dependent AcCoA hydrolysis.

### Statistics

Weights were compared over time by a two-way analysis of variance followed by Bonferroni post hoc test. Latency and incidence of first palpable mammary tumors over time were tested by logrank (Mantel-Cox) test. Mann-Whitney tests were used to compare mammary tumor multiplicity. Terminal tumor incidence was tested by a Fischer’s exact test. Enzymatic activities and endogenous AcCoA concentrations were compared by unpaired t-tests. All statistical tests were done using Prism software by GraphPad (La Jolla, CA). Data are displayed and presented as the average ± standard error of mean (SEM) for each respective group.

## Results

### Weight gain

**Fig. 2:**
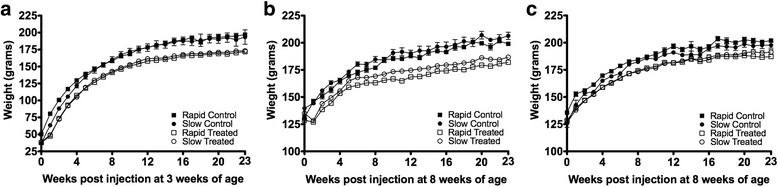
Weekly body mass (mean grams ± SEM) of rapid and slow acetylator *Nat2* congenic rats post administration of specified carcinogen or vehicle control. In all three panels squares represent rapid acetylator and circles represent slow acetylator congenic rats. Filled-shapes represent vehicle-treated and unfilled-shapes represent. MNU or DMBA-treated. **a** Weights for rats treated with MNU at 3 weeks of age. The difference between weights of the vehicle-treated and MNU-treated is statistically significant by two-way ANOVA with Bonferroni post hoc test (*p* < 0.05). **b** Weights for rats treated with MNU at 8 weeks of age. The weights of the vehicle-treated and MNU treated rats were statistically significant by two-way ANOVA with Bonferroni post hoc test (*p* < 0.05). **c** Weights for rats administrated DMBA at 8 weeks of age. The weights of the vehicle-treated and DMBA-treated rats were not statistically significant by two-way ANOVA with Bonferroni post hoc test (*p* > 0.05). In all panels, weights of MNU/DMBA-treated rats were not significantly different between rapid and slow NAT2 acetylator rat strains

Rapid and slow acetylator congenic rats administered vehicle at either 3 or 8 weeks demonstrated normal weight gain. Initially, all MNU-treated rats experienced hindered weight gain post administration, but then recovered to a rate of weight gain similar to vehicle-treated rats. MNU-treated rats never reached the same weight as the vehicle-treated counterparts (Fig. 2). No statistically significant (*p* > 0.05) differences in weight gain were noted between MNU-treated rapid and slow acetylator congenic rats.

DMBA-treated rats displayed no initial hindrance in weight gain; however, during the time following DMBA administration, DMBA-treated rats tended to have lower body weight than vehicle-treated rats (Fig. 2). No statistically significant (*p* > 0.05) differences in weight gain were noted between DMBA-treated rapid and slow acetylator congenic rats.

### Palpable mammary tumor latency and incidence

**Fig. 3:**
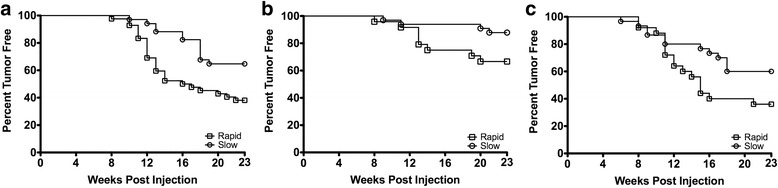
Kaplan-Meier plot of palpable mammary tumors in congenic rats. Onset of tumors in rats exposed to **a** MNU at 3 weeks of age, **b** MNU at 8 weeks of age, and **c** DMBA at 8 weeks of age. Squares illustrate rapid and circles illustrate slow acetylator *Nat2* congenic rats. Rapid acetylator congenic rats had a significantly (*p*<0.05) shorter latency for development of first palpable mammary tumor compared to slow acetylator congenic rats after MNU administration at either 3 or 8 weeks of age.﻿ Similar findings were obtained after DMBA administration at 8 weeks of age although the difference was not significant (*p*=0.065)

As shown in Fig. 3, rapid acetylator congenic rats had a significantly (*p*<0.05) shorter latency for development of their first palpable mammary tumor compared to slow acetylator NAT2 congenic rats after administration of MNU in females treated at either 3 or 8 weeks of age.

DMBA-treated rapid acetylator rats displayed a higher tumor incidence over the course of weeks than DMBA-treated slow acetylator rats, although logrank test analysis provides a *p*-value (*p* = 0.065) slightly greater than our established *p*-value threshold of significance (*p* = 0.050).

### Terminal mammary tumor incidence and multiplicity

As shown in Table 1, female congenic rats, administered MNU at 3 weeks of age, developed tumors in 66.7% of the rapid acetylator congenic rats with an average 1.00 ± 0.17 tumors per rat. In contrast, tumors were found in 52.9% of slow acetylator congenic rats with an average of 0.67 ± 0.12 tumors per rat. These mammary tumor incidence and multiplicity results were not significantly different between rapid and slow acetylator rats administered MNU at 3 weeks of age.

**Table 1:** Mammary tumor totals in rapid and slow acetylator congenic rats administered MNU or DMBA

Treatment Protocol	Acetylator phenotype	Rats treated and necropsied	Rats with tumors (%)^a^	Total tumors	Total tumors per rat^b,c^	Total tumors per rat in tumor- bearing rats^c^
MNU 3 weeks	Rapid	42	28 (66.7%)	42	1.00 ± 0.17	1.50 ± 0.20
MNU 3 weeks	Slow	34	18 (52.9%)	23	0.67 ± 0.12	1.28 ± 0.11
MNU 8 weeks	Rapid	24	10 (41.6%)*	12	0.50 ± 0.14*	1.20 ± 0.20
MNU 8 weeks	Slow	33	5 (15.2%)*	8	0.24 ± 0.12*	1.60 ± 0.24
DMBA 8 weeks	Rapid	25	19 (76.0%)*	30	1.20 ± 0.16**	1.58 ± 0.12
DMBA 8 weeks	Slow	30	13 (43.3%)*	17	0.57 ± 0.14**	1.31 ± 0.17

^a^Tumor incidence

^b^Tumor multiplicity

^c^Mean ± SEM. Total mammary tumors differed significantly *(*p* < 0.05) ** (*p* < 0.01) between rapid and slow acetylator rats

As shown in Table 1, female congenic rats administered MNU at 8 weeks of age, developed mammary tumors in 41.6% of rapid acetylators with 0.50 ± 0.14 average tumors per rat. Slow acetylators developed mammary tumors in 15.2% of those given MNU, with 0.24 ± 0.12 tumors per rat. Thus, rapid acetylator rats exhibited significantly higher mammary tumor incidence (*p* = 0.035) and multiplicity (*p* = 0.050) compared to slow acetylator rats.

No other tissue/organ tumors besides mammary tumors were observed in either MNU- or vehicle-treated rats. A few rats displayed discoloration of the eyes, thought to be an onset of cataracts. Cataracts and retinal degradation can occur in rats following MNU administration [45].

As shown in Table 1, female congenic rats exposed to DMBA developed mammary tumors in 76.0% of rapid acetylators with 1.20 ± 0.16 average tumors per rat. Slow acetylators developed mammary tumors in 43.3% of those given DMBA, with 0.57 ± 0.14 tumors per rat. Rapid acetylator rats had significantly higher mammary tumor incidence (*p* = 0.027) and multiplicity (*p* = 0.004) compared to slow acetylator rats. No other tissue/organ tumors besides mammary tumors were observed in DMBA- or vehicle-treated rats.

### Pathology

**Fig. 4:**
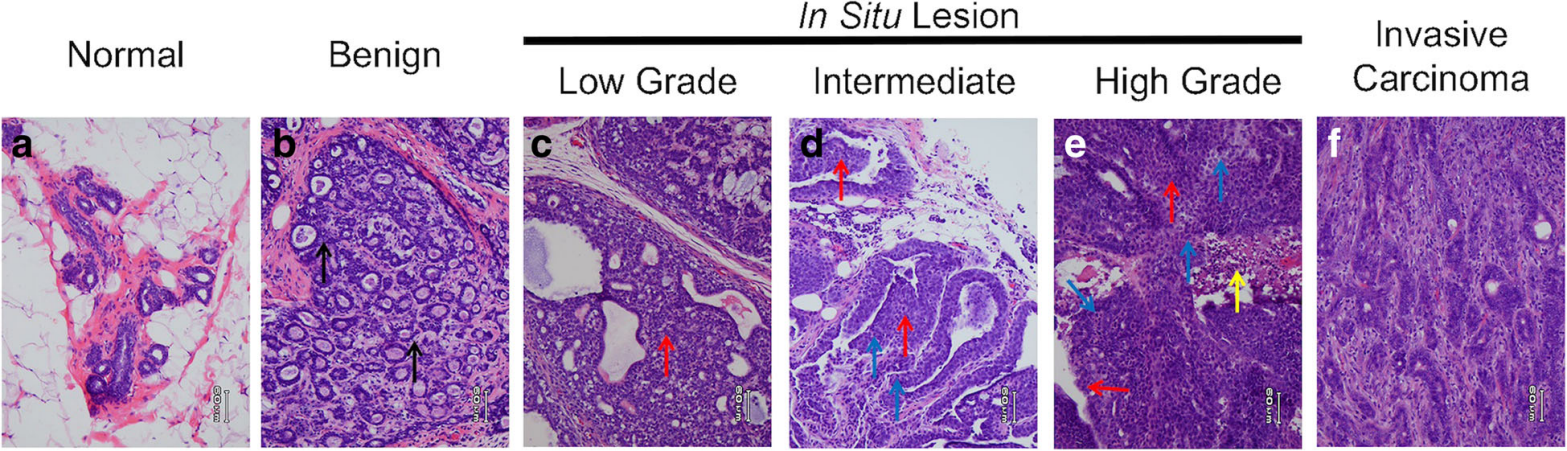
Photomicrographs of H&E stained slides depicting the criteria utilized to classify tumors. **a** Normal mammary tissue. **b** Benign tumor showing proliferative disease with no cytologic atypia and minimal to no overgrowth of the epithelial or myoepithelial component (*black arrows*). **c**–**e** *CIS* lesions with overgrowth of the epithelial and/or myoepithelial component (*red arrows*). **c** Low grade *CIS* lesion with low grade cytologic atypia and occasional mitoses or single cell apoptosis. **d** Intermediate grade *CIS* lesion with intermediate to high grade cytologic atypia with increased mitoses (*blue arrows*) or single cell apoptosis. **e** High grade *CIS* lesion with intermediate to high grade cytologic atypia with tumor necrosis (*yellow dashed arrow*) and increased mitoses (*blue arrows*). **f** Invasive carcinoma with glands or single cells infiltrating through stroma

A majority of the mammary tumors that developed in carcinogen-treated rats were classified as non-invasive (Fig. 4). More tumors classified as benign were noted in rats administered MNU at 3 weeks compared to 8 weeks of age (15% and 0%, respectively).

Among the rapid and slow congenic rat strains administrated MNU at 3 weeks of age, ~11% of tumors were classified as invasive carcinomas in the rapid acetylators (3/28) versus none in the slow acetylators (0/18). Low grade *CIS* tumors were the most common histomorphological classification of tumors in both rapid and slow rat *Nat2* congenic strains.

Rats administered MNU at 8 weeks of age exhibited similar percentages of mammary tumors classified as low grade *CIS* in rapid (5/10) and slow (2/5) acetylators. Mammary tumors classified as high grade *CIS* tumors were observed in 3 rapid acetylator congenic rats (3/10), and the only tumor classified as invasive was found in one slow acetylator rat (1/5).

In general, rats administered DMBA displayed a very similar distribution pattern of tumor pathology. The rapid NAT2 congenic rats had more benign tumors (4/19 or 21%) than slow NAT2 congenic rats (1/13 or 8%). The slow congenic rats had a higher percentage of tumors with an invasive classification than the rapid congenic rats (31% [4/13] vs 11% [2/19]).

### Enzymatic activities of recombinant rapid and slow rat NAT2

**Fig. 5:**
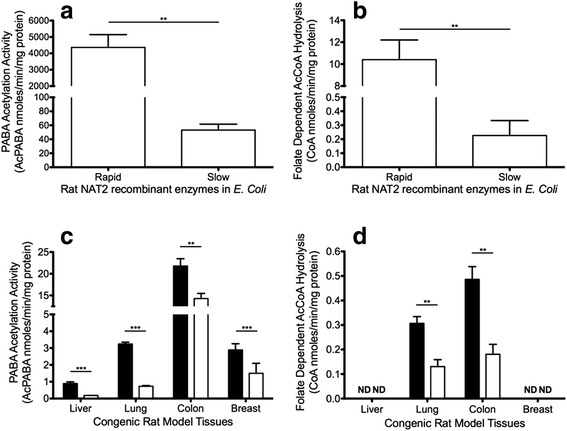
Rat NAT2 PABA acetylation and folate-dependent AcCoA hydrolysis activity. **a**, **c** PABA acetylation activity. **b**, **d** Folate-dependent AcCoA hydrolytic activity. **a**, **b** Triplicates from the same recombinant lysates. **c**, **d** Lysates of individual tissues collected from 5 rapid (*closed bars*) or 5 slow (*open bars*) acetylator NAT2 F344 congenic rats. “ND” = not detectable. Differences between rapid and slow acetylator rats differed significantly ***p < 0.001; **p < 0.01

PABA *N*-acetylation velocities for rat arylamine *N*-acetyltransferase 2 catalyzed by recombinant rapid acetylator NAT2 was significantly (*p* = 0.005) higher than by recombinant slow acetylator NAT2 (Fig. 5a). The folate-dependent AcCoA hydrolysis catalyzed by rat NAT2 was significantly (*p* = 0.005) higher for recombinant rapid acetylator NAT2 than recombinant slow acetylator NAT2 (Fig. 5b).

### Rapid and slow acetylator rat NAT2 activities in tissue lysates

The PABA *N*-acetylation activities of *Nat2* congenic rats measured in lysates from several tissue-types was significantly higher in rapid than slow acetylator liver (*p* < 0.001), lung (*p* < 0.001), colon (*p* = 0.007), and mammary gland (*p* < 0.001) (Fig. 5c). The folate-dependent AcCoA hydrolysis was significantly higher in rapid acetylator than slow acetylator lung (*p* = 0.002) and colon (*p* = 0.002) (Fig. 5d). No detectable folate-dependent AcCoA hydrolysis activity was observed in liver and mammary tissue lysates (threshold of detection 0.05 nmoles/min/mg protein).

### NAT2 activity and endogenous AcCoA concentrations in isolated congenic REFs

**Fig. 6:**
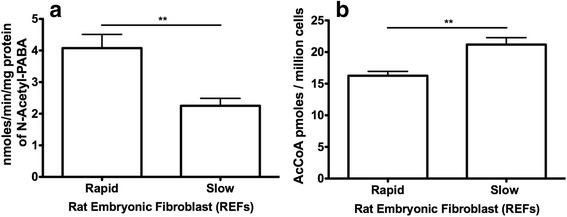
NAT2 activity and AcCoA level measured in rat embryonic fibroblasts (REFs) from rapid and slow rats. **a** Rapid NAT2 REFs (*N* = 7) have a higher level of PABA acetylation activity than slow NAT2 REFs (*N* = 9). **b** The amount of AcCoA per million cells is lower in rapid NAT2 REFs (*N* = 8) than in slow NAT2 REFs (*N* = 10). **Differs significantly between rapid and slow acetylators (*p* < 0.01)

The REFs isolated from the congenic rat strains were characterized for their ability to acetylate PABA and their endogenous AcCoA concentrations after the fourth passage. PABA N-acetylation in rapid NAT2 REFs was significantly higher than in slow NAT2 REFs (*p* = 0.002) (Fig. 6a). Endogenous AcCoA concentrations levels in the rapid NAT2 REFs were lower compared to that of the slow NAT2 REFs (*p* = 0.003) (Fig. 6b).

## Discussion

This study is the first to utilize a functional animal model to look at tumorigenic differences between rapid and slow rat NAT2 activity using carcinogens that do not require NAT2 activity for activation/deactivation. This functional animal model mimics human populations. Rat NAT2 is the ortholog of human NAT1. Genetic variants and other factors influence human NAT1 activity levels. The NAT1*10 haplotype is associated with increased NAT1 activity in human tissue samples [15–17]. Other polymorphic haplotypes encode a less stable NAT1, leading to a decrease in enzymatic activity. Our results in Nat2 congenic rats suggest that human genetic variation resulting in differential NAT1 activity may influence human breast cancer. Furthermore, our findings suggest that NAT1*10 human haplotypes may have a higher risk of breast cancer compared to the reference NAT1*4 haplotype. Also, polymorphic variants that decrease NAT1 activity would likely have a reduced cancer risk.

In the present study, we investigated whether chemical carcinogens induced tumors more rapidly or in greater abundance in rapid than slow *Nat2* congenic rat strains. MNU was administered at either a pre-pubescent age (3 weeks) or a post-pubescent age (8 weeks). Additionally DMBA was administered at only post-pubescent age (8 weeks). We observed decreased latency and increased incidence of palpable mammary tumors in congenic rapid acetylator rats than in congenic slow acetylator rats treated with either MNU or DMBA. Terminal multiplicity and incidence also were higher in rapid than slow acetylator rats administered MNU or DMBA.

The role of human NAT1 in cancer is not well understood, but the current understanding of NAT1’s potential importance in cancer is expanding. Several microarray studies have shown that elevated NAT1 expression is correlated with estrogen receptor positive (ER+) breast cancer samples [5, 6, 46]. Human NAT1 expression in breast cancer is predicted to be a valuable indicator for antiestrogen responsiveness [47] and an indicator of a positive prognosis, particularly in ER+ breast cancer [48]. Normal human mammary luminal epithelial cell lines (HB4a) engineered to overexpress NAT1 continue growing compared to normal HB4a cells that reach a growth plateau [5]. The present study adds to the developing and complex narrative of NAT1 in cancer, by providing the first animal model data that show an association between NAT1 activity levels and tumorigenesis independent of carcinogen metabolism.

Rapid acetylator rats displayed elevated risk of mammary tumors compared to slow acetylator MNU-treated rats regardless of whether treatment occurred when immature or mature. Pre-pubescent exposure to MNU, regardless of NAT2 activity phenotype, resulted in greater palpable tumor incidence, as well as increased terminal mammary tumor incidence and multiplicity (compare panels in Fig. 3). This increase with earlier age of MNU-exposure is consistent with the known elevated breast cancer susceptibility of prepubescent female breasts to cancer induced by ionizing radiation [49]. MNU, a simple alkylating agent, is radiomimetic [50]. Humans are exposed to alkylating chemical carcinogens in diet, tobacco products, cosmetics, drugs, and chemotherapy [51]. The results reported in the current study suggest that the human NAT1 (an ortholog of rat NAT2) phenotype and prepubescent carcinogen exposure should be studied as additional factors in human breast cancer susceptibility.

The role higher rat NAT2 activity plays in increased tumorigenesis is poorly understood. Our hypothesis is that a more active rat NAT2 might lower vital compounds needed for DNA repair to a greater extent than a less active rat NAT2. Rat NAT2 and human NAT1 are orthologous enzymes that, in addition to their acetylation capacity, catalyze the hydrolysis of AcCoA to acetate and coenzyme A in the presence of folate [43, 52]. Thus, rapid rat NAT2 (or more active human NAT1) can reduce intracellular levels of AcCoA to a greater extent than slow rat NAT2, affecting crucial pathways supporting DNA repair (e.g. histone and p53 acetylation). In support of this hypothesis, measurement of NAT2 activity in rapid acetylator rat tissues showed higher levels of PABA N-acetylation and folate-dependent AcCoA hydrolysis than in slow acetylator rat tissues. These differences were also reflected in recombinant rapid and slow acetylator NAT2 expressed in bacteria. As shown with congenic rat tissues and recombinant NAT2 proteins, REFs isolated from rapid NAT2 rats had a higher PABA N-acetylation activity level than REFs isolated from the slow NAT2 congenic rats. Furthermore, rapid NAT2 REFs had lower levels of AcCoA compared to the slow NAT2 REFs. This observation parallels the findings with folate-dependent AcCoA hydrolysis, in that rapid acetylator NAT2 is able to hydrolyze AcCoA at greater rates than slow acetylator NAT2 and thus more likely to reduce endogenous AcCoA levels.

The study of intracellular AcCoA concentration levels has not been well characterized after DNA insult. Some studies have shown AcCoA concentrations control cell growth, cellular autophagy, and histone acetylation [53–55]. Other studies have shown reduced acetylation of downstream targets if acetyl-CoA carboxylase, ATP-citrate lyase, or pantothenate kinase are knocked out [55–57]. Future studies will further explore human NAT1 activity in relation to intracellular AcCoA concentrations and associated differences in acetylation targets. Unpublished data from our laboratory suggests that human NAT1 knockout cancer cell lines have elevated AcCoA concentrations compared to their wildtype counterparts.

Breast cancer is one of the most common types of cancer. It is estimated that in 2016 breast cancer will be the most diagnosed type of cancer in United States women, excluding basal and squamous cell skin cancers [58]. It is also expected that breast cancer will be one of the leading causes of death from cancer in women, with only lung cancer ranking higher [58]. Breast cancer’s persistence as one of the most formidable cancers in women illuminates the need to understand differences in cancer susceptibility and discover novel avenues for prevention and treatment. The studies in this paper primarily focus on breast cancer. However given ubiquitous expression of NAT1, the findings here are likely to be observed in other cancer sites such as lung, colon, urinary bladder, and prostate.

## Conclusions

In summary, we report differences in mammary tumor incidence and multiplicity between rapid and slow acetylator congenic F344.WKY-*Nat2* rats following administration of a direct acting carcinogen (MNU) and pro-carcinogen (DMBA). Rat NAT2 does not metabolize these carcinogens. We have shown that folate-dependent AcCoA hydrolysis is higher in rapid compared to slow acetylator rat NAT2, both in recombinant form and in tissue lysates from multiple organs. Additionally, we provided the first evidence that rat NAT2 plays a role in modulating intracellular AcCoA concentrations. Our findings suggest that further study of a role for human NAT1 in cancer susceptibility apart from the role of NAT1 in carcinogen metabolism is warranted.

## Abbreviations

AcCoA: Acetyl coenzyme A
C: Degree Celsius
*CIS*: *Carcinoma* In Situ
DMBA: 7,12-dimethylbenzanthracene
g: Gravity
HPLC: High-performance liquid chromatography
hrs: Hours
IP: Intraperitoneal
kg: Kilogram
M: Molar
mg: Milligram
mins: Minutes
ml: Milliliter
mm: Millimeter
mM: Millimolar
MNU: Methylnitrosourea
NAT1: Human arylamine N-acetyltransferase 1
NAT2: Rat arylamine N-acetyltransferase 2
nm: Nanometer
nmoles: Nanomoles
PABA: *para*-aminobenzoic acid
PBS: Phosphate-buffered saline
pmoles: Picomoles
REFs: Rat embryonic fibroblasts
μg: Microgram
μl: Microliter
μm: Micrometer
μM: Micromolar
